# Renal collecting duct carcinoma with extensive coagulative necrosis mimicking anemic infarct: report of a case and the literature review

**DOI:** 10.1186/1746-1596-8-119

**Published:** 2013-07-16

**Authors:** Qinqin Xu, Qinghua Cao, Ni Liu, Ziwen Fang, Ziyin Ye, Tingsheng Peng

**Affiliations:** 1 Department of Pathology, The First Affiliated Hospital of Sun Yat-sen University, No. 58, Zhongshan Road II, Guangzhou 510080, China; 2 Department of Diagnostic Radiology, The First Affiliated Hospital of Sun Yat-sen University, No. 58, Zhongshan Road II, Guangzhou 510080, China; 3 Department of Pathology, Guangming New District People’s Hospital, Shenzhen 518106, China

**Keywords:** Collecting duct carcinoma (CDC), Renal epithelial tumors, Extensive coagulative necrosis, Anemic infarct, Differential diagnosis

## Abstract

Collecting duct carcinoma (CDC) with a mass of coagulative necrosis is very rare. We report here a case of CDC with extensive geographic coagulative necrosis mimicking anemic infarct with tumor cells embedded around the necrotic foci in a 73-years-old man. Histopathological examination showed that tumor nests near the necrotic foci were arranged as angulated tubules, tubulopapillary and glandular structures. Neoplastic cells had moderate to abundant eosinophilic cytoplasm and large hyperchromatic nuclei with prominent nucleoli as Fuhrman nuclear grade 3 or 4. The tumor cells were positive for pan-Cytokeratin, Vimentin, E-cadherin, CD10, and CK7, confirming the diagnosis as CDC. The patient is still alive 6 months later from nephrectomy, a long time following up is needed to learn the prognosis. Conclusively, morphology from different portions of the lesion, immunohistochemical stain and the combination analysis of the radiological features is essential to make a precise pathological diagnosis of CDC. And CDC should also be distinguished from clear cell renal cell carcinoma, renal medullary carcinoma, urothelial carcinoma with glandular differentiation, renal neuroendocrine tumor, renal epithelioid angiomyolipoma, renal pigmented paraganglioma and renal mesenchymal chondrosarcoma etc.

**Virtual Slides:**

The virtual slide(s) for this article can be found here: http://www.diagnosticpathology.diagnomx.eu/vs/1264270525975030

## Background

Adult renal tumors comprise a range of distinct clinicopathologic subtypes with differing clinical and/or syndrome associations, gross, microscopic, immunohistochemical characteristics. Collecting duct carcinoma (CDC) of the kidney is an unusual variant of renal cell carcinoma. It originates from the epithelium of the collecting tubule and accounts for less than 1% of the incidence of renal epithelial neoplasms [1]. CDC was firstly reported by Foot and Papanicolaou in 1949 [2]. And it was formally recognized as an unique clinicopathologic subtype of RCC following the report and description of 6 new cases by Fleming and Lewi in 1986 [3].

Unlike the majority of renal cell carcinomas, CDC is characterized by distinct clinicopathological features, aggressiveness and poor prognosis [1]. A male to female ratio of approximately 2:1 with a mean age of occurrence in the sixth decade [1]. Grossly, the tumor is often centrally located in or near the region of the renal pelvis and appears gray or white without extensive hemorrhage [4,5]. The tumor often has irregular infiltrative borders and no extensive hemorrhage and necrosis [5,6]. The most common architectural patterns of CDC include angulated tubules or tubulopapillary structures and glandular structures [6]. High-grade nuclear features with pleomorphism and prominent eosinophilic nucleoli as Fuhrman nuclear grade 3 or 4 are common [7]. Occasionally, focal necrosis were noted in the foci [5]. In contrast to previously cases, we present here a CDC case with extensive necrosis and cystic formation in an old male patient, which were seldom observed in this type tumor of kidney. The clinicopathological features of this case and the differential diagnosis was also been discussed.

## Case presentation

### Clinical history

**Figure 1:**
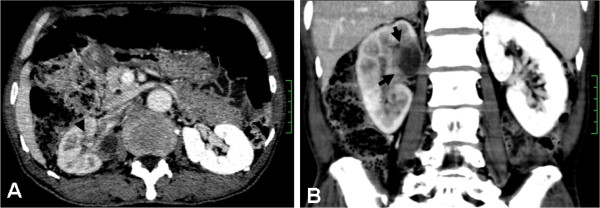
**CT images.** (**A**) Axial contrast-enhanced nephrographic-phase CT image obtained at level of renal hilum shows an ill-defined, heterogeneous mass with central cystic change involving the parenchyma of the right kidney, with extension into the renal pelvis and right vein. (**B**) Reconstructed coronal contrast-enhanced CT image obtained during the excretory phase shows the compression and distortion of the renal pelvis.

A 73-year-old man was hospitalized because of right flank pain. There was no history of cigarette smoking and no family history of malignancy. No history of occupational exposure to carcinogens and chronic diseases and chronic medications was obtained. On admission the patient appeared to have good general condition without fever, weight loss and dyspnoea. Physical and neurological examinations showed no abnormality. The laboratory results including blood count and classification, liver and renal function, and tumor markers were within the normal range. Computed tomography showed a 2.5×3.4 cm complex solid/cystic mass in the upper right kidney invading renal sinus and peri-renal tissue, which was an ill-defined, heterogeneous mass with central cystic formation (Figure 1, Arrow showed). Chest and abdominal x-ray were normal for lung and other abdominal organs. A preoperative presumed diagnosis was renal coagulative necrosis or tumor and a radical right nephrectomy was performed.

## Material and methods

The specimen was fixed in a 10% neutral formalin solution and embedded in paraffin. Four micrometer-thick sections were prepared and stained with hematoxylin-eosin. An Envision two-step assay was used for the immunohistochemistry staining. Commercially available monoclonal antibodies were employed: Pan Cytokeratin (Mouse mAb (AE1/AE3); 1:200), Vimentin (Mouse mAb (V9);1:1000), CD10 (Mouse mAb (56C6); 1:200), E-Cadherin (Mouse mAb(NCH-38);1:200), CK7(Mouse mAb(OV7L12/30);1:200), CK20 (Mouse mAb(Ks20.8);1:200), CD31(Mouse mAb(JC70A);1:200), CD34 (Mouse mAb(QBEnd10); 1:200), Actin (MousemAb(1A4); 1:200), CD56 (Mouse mAb(123C3); 1:200), CgA (Rabbit pAb; 1:200), MelanA (Mouse mAb(A103); 1: 200), HMB45 (Mouse mAb(HMB45); 1: 200), Ki-67 (Mouse mAb(MIB-1);1:200). All the primary antibodies and HRP-conjugated secondary antibodies were obtained from DAKO Inc., Denmark.

### Pathological findings

**Figure 2:**
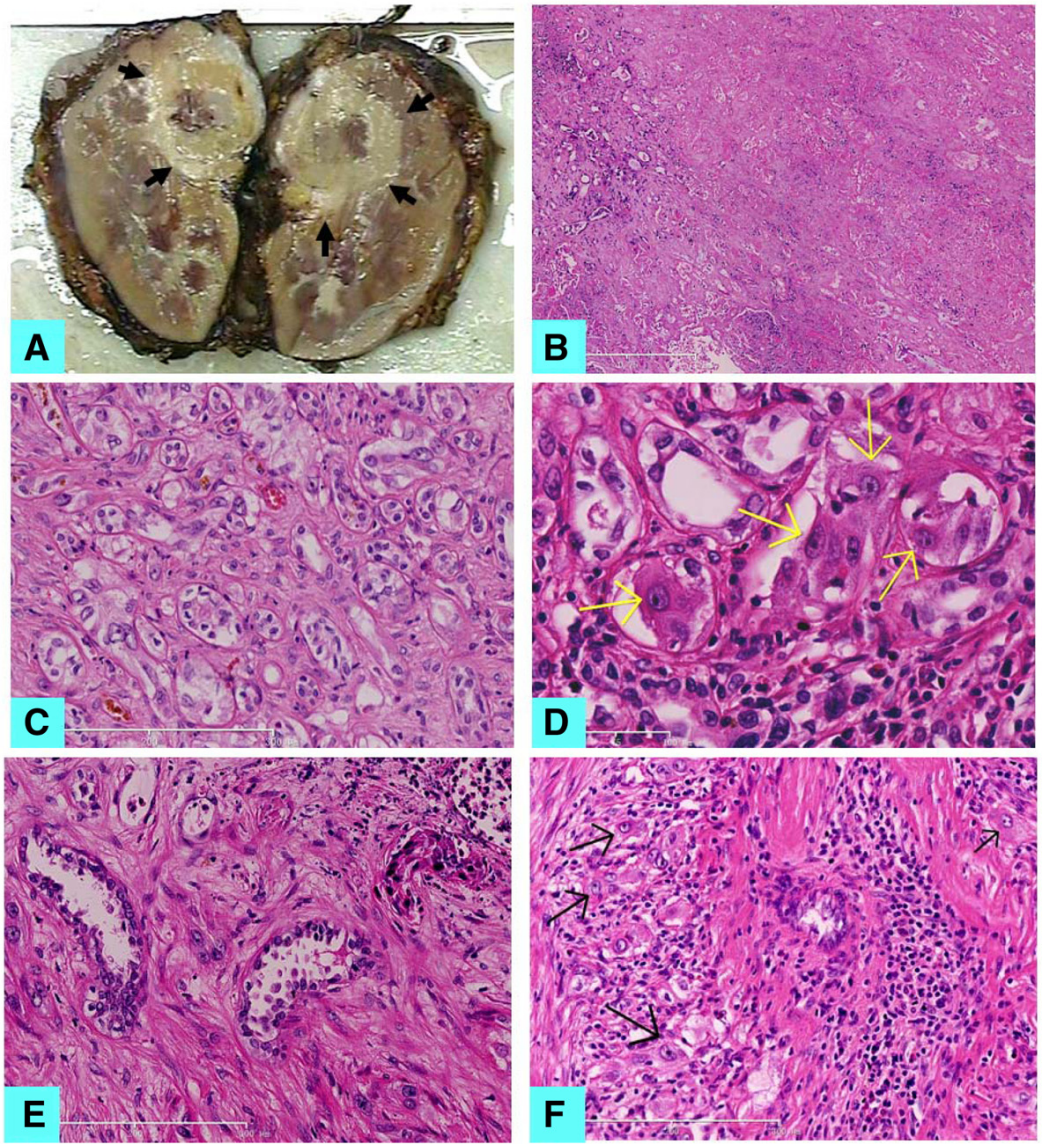
**Morphology of this renal tumor.** (**A**) Macroscopic appearance of the tumor. A global firm grayish yellowish tumor in the upper part of kidney and near hilus renalis, involving the renal medulla and the cortex. (**B**) Histological feature of the tumor showed large areas of geographic coagulative necrosis of kidney (H&E × 100). (**C**) Tubules or angulated glands irregularly infiltrating the renal parenchyma with a prominent interstitial growth pattern (H&E × 200). (**D**) Tumor cells with abundant eosinophilic cytoplasm and large hyperchromatic nuclei with prominent nucleoli (H&E × 400). (**E**) Hobnail nuclei were noted in the fibrous background (H&E × 400). (**F**) Microabscess like necrosis was occasionally seen with scattered atypical cells (H&E × 400).

Gross examination of the kidney revealed a kidney measuring 10.0 × 7.5 × 5.5 cm. On cut section of the specimen, a 2.5 × 2.5 × 2.0 cm pale mass was mainly found in the upper part of kidney and near hilus renalis. The mass appeared as a firm grayish yellowish, solid and cystic nodule with irregular borders and extensive geographic coagulative necrosis, infiltrating renal sinus and peri-renal fat (Figure 2A).

Histological examination revealed extensive geographic coagulative necrosis with the contour of the necrotic tissues and a large amount of debris of the dead cells. This image was firstly considered as kidney anemic infarct by some pathologists. A few atypical cell nests embedded in the edge of the necrotic foci and the wall of the cyst were carefully found, which caused the caution as a tumor (Figure 2B). More 10 samples taken from the whole rest mass had been analyzed, and there were much more tubules or angulated glands irregularly infiltrating the renal parenchyma with a prominent interstitial growth pattern, which were interweaved with the normal kidney tubules around the necrotic foci (Figure 2C). It was confirmed to be a malignant tumor that many cells in the irregular glands showed moderate to abundant eosinophilic or clear cytoplasm and large hyperchromatic nuclei with prominent nucleoli, which were of Fuhrman nuclear grade 3 or 4. (Figure 2D). On rare occasions, Hobnail nuclei were noted in the fibrous background (Figure 2E). Microabscess like necrosis was occasionally seen with scatted atypical cells (Figure 2F). The desmoplasia was accompanied by a predominantly lymphocytic or occasionally mixed inflammatory infiltrate including neutrophils and eosinophils.

**Figure 3:**
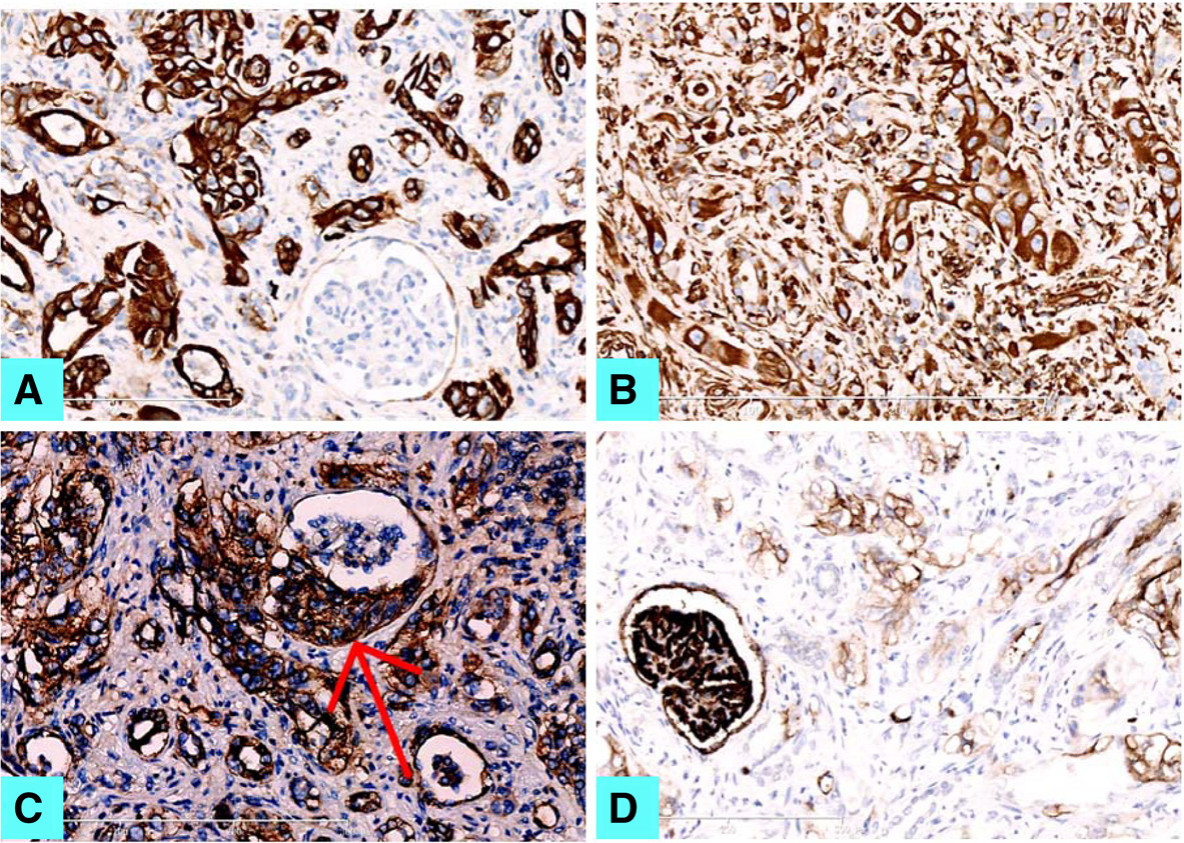
**Immunohistochemistry staining for the invasive tumor.** (**A**) pan-CK, (**B**) Vimentin, (**C**) E-Cadherin and (**D**) CD10 staining (IHC × 200).

Immunohistochemical staining showed atypical cell nests were positive for both CK (Figure 3A) and Vimentin (Figure 3B), partly positive for E-cadherin (Figure 3C), and weakly positive for CD10 (Figure 3D) and CK7 (not shown). They were also negative for CD31, CD34, CD56, CgA, Actin, Melan-A, HMB-45, and CK20. MIB was up to 20% in the tumor cells. Immunohistochemical staining helped to find the involvement of the glomeruli and the vascellum by malignant tumor cells. Based on these histological and immunohistological findings, this lesion was coincident with the diagnosis as collecting duct carcinoma (CDC) of Bellini of the kidney, not anemic infarct.

## Discussion

Collecting duct carcinoma (CDC) of the kidney is a rare renal cell carcinoma. Occasionally, focal necrosis were noted in the foci [5], while CDC with extensive coagulative necrosis is very rare. In this case, because of the extensive geographic coagulative necrosis, it was easily to be misdiagnosed as renal anemic infarct at the first sight.

As known, kidney anemic infarcts tend to be wedge-shaped foci with the occluded vessel at the apex and the periphery of the kidney forming the base, and there are no cellular atypia in the surrounding normal tissue [8]. In this case, with sufficient resections from different portions, the atypical tumor cells were seen to infiltrate the upper and middle renal calyxes, and involve the glomeruli and the vascellum. The coagulative necrosis foci was also different from infarct either in shape or the characteristics. The difference between CDC and kidney anemic infarct was shown in Table 1. Immunohistochemical staining showed that the tumor cells were positive for CK, Vimentin, partly positive for E-cadherin, weakly positive for CD10 and CK7, which was basically consistent with the literature [9]. After the exclusion of the other tumors of kidney, we made the final diagnosis as kidney collecting duct carcinoma.

**Table 1:** Comparison of the morphological difference between kidney CDC in this case and anemic infarct of kidney

	**Collecting duct carcinoma (CDC) of kidney in this case**	**Anemic infarct of kidney**
Gross morphology	A firm grayish yellowish, solid and cystic nodule with irregular borders and extensive geographic coagulative necrosis	One or more wedge-shaped necrotic foci, with the occluded vessel at the apex and the periphery of the kidney forming the base
Histological features	Atypical tubules or angulated glands around the coagulative necrotic foci, irregularly infiltrate the renal parenchyma with a prominent interstitial growth pattern; Tumor cells have moderate to abundant eosinophilic or clear cytoplasm and large hyperchromatic nuclei with high nuclear grade	Coagulative necrosis with the contour of the necrotic tissue with no cellular atypical features

The diagnostic criteria of CDC have not yet been fully established, it is often difficult and, to some extent, is one of exclusion (See Table 2 in detail). CDC is an aggressive tumor, sometime with extensive multi-organ metastasis including the lung, liver, spleen, bone marrow, adrenal gland, para-aortic lymph node, proximal ureter, and meninges, resulting in complex presentation [10,11]. Therefore the prognosis of CDC is poor, with approximately 70% of patients dying of disease progression within 2 years after diagnosis [12,13]. In this case, the patient is still alive 6 months later from nephrectomy, so that a long time following up is needed to judge the exact prognosis.

**Table 2:** Comparison of the immunohistochemical features of kidney CDC and other differential diagnosis

**Tumors**	**Clinical features**	**Histopathology**	**Immunohistochemistry**	
			**Positive**	**Negative**
CDC	A male to female ratio of approximately 2:1 with a mean age of occurrence in the sixth decade	Angulated tubules or tubulopapillary and glandular structures associated with a desmoplastic stroma; Large irregular tumor cells with abundant eosinophilic or clear cytoplasm and large hyperchromatic nuclei with prominent eosinophilic nucleoli	CK(AE1/AE3) Vimentin E-cadherin CK7 CD10(weakly)	CK20
CCRCC	Occurs in adults (average 70 years), often associated with VHL disease. The tumor are typically multi-focal and bilateral	Solid, alveolar and acinar patterns, containing a network of small thin-walled blood vessels. The tumor cells have abundant clear cytoplasm with round and uniform nuclei	CD10 CK Vimentin	E-cadherin CK7 CK20
Renal medullary carcinoma	A rare tumor. Occurs in younger cohort mainly in the third decade of life, all the patients had sickle cell trait and hemoglobin abnormalities	Poorly differentiated tumor cells arrange as sheets and reticular pattern. The cells are eosinophilic with clear nuclei and prominent nucleoli, often mixed with neutrophils and lymphocyte at the margin	CK EMA CEA	Vimentin
Urothelial carcinoma with glandular differentiation or Urothelial adenocarcinoma	Less common type of urothelial carcinoma. A tumor with mixed glandular and urothelial differentiation is definited	True glandular spaces present, tubular or enteric glands with mucin secretion. Tumor cells have moderate to abundant cytoplasm and large hyperchromatic nuclei	CK CK7 CK20 (in most adenocarcinoma)	Vimentin CD10
NET	Rare renal tumor. Occurs in adults (average 60 years), with no sex predilection	Trabecular nests or solid nests. Largely polygonal tumor cells with granular clear cytoplasm and round to oval uniform nuclei with rare mitotic figures	CK CgA Syn NSE (weakly)	Vimentin Desmin
Epithelioid AML	More than a half have a history of tuberous sclerosis; both sexes are equally affected, the mean age is 38 years	Round to polygonal tumor cells arranged in sheets, with abundant granular cytoplasm and enlarged vasicular nuclei often with prominent nucleoli	Melan-A HMB-45 Actin (partly)	CK CK7 E-cadherin
Pigmented paraganglioma	Rare renal tumor. Most tumor are small, occurs in adults	Tumor cells arranged in nests (Zellballen), delicate fibrovascular stroma, large round or polygonal tumor cells with round or oval nuclei	CD56 CgA Syn S-100 (sustentacular cells positive)	CK CK8/18 Vimentin CD10 Actin
Mesenchymal chondrosarcoma of kidney	Very rare renal tumor	Undifferentiated spindle to oval shaped cells, and islands of cartilage	S-100 CD99 Vimentin	CK EMA E-cadherin

Because there were tumor areas composed with clear cells, clear cell renal cell carcinoma (CCRCC) should be eliminated too. CCRCC is a typically globular tumor which commonly protrude from the renal cortex as a round mass, tending to be well-circumscribed expansile lesions invariably with a pseudocapsule. It is in contradistinction to the infiltrative nature of collecting duct carcinoma [1]. Immunohistochemical staining was distinguishable to show that tumor cells of CCRCC diffusely positive for CD10, vimentin and negative for E-cadherin, CK7, CK20, while that of CDC are positive for E-cadherin, CK7, vimentin, partially CD10, and negative for CK20 [9].

Renal medullary carcinoma is another distinctive tumor needed to be distinguished with CDC. Renal medullary carcinoma occurs in younger cohort mainly in the third decade of life, with a strong male predominance (10:1) individuals of African ancestry, and all the patients had hemoglobin abnormalities and much shorter median survival [1,14]. There is marked overlap on clinical features, histology, immuno-phenotype, patterns of metastases, and uniformly aggressive outcome between collecting duct carcinoma and renal medullary carcinoma, suggesting a close interrelationship between them. By some views, renal medullary carcinoma is considered as a distinctive clinicopathologic subtype within the aggressive category of carcinoma of collecting ducts of Bellini [6].

Urothelial carcinoma of the renal pelvis with glandular differentiation or urothelial adenocarcinoma invading the renal parenchyma may elicit desmoplastic response, thus resembling collecting duct carcinoma. In this case, the tumor cells were positive for both CK and vimentin, and weakly positive for CD10. Because urothelial carcinoma cells are positive for CK, not for vimentin and CD10, it was not so difficult to make proper diagnosis with immunohistochemical markers [1].

Renal primary neuroendocrine tumor (NET) also have trabecular nests or solid nests. The tumor cells were largely polygonal with clear cytoplasm and indistinguishable cytoplasmic boundaries [15]. Sometime the large polygonal tumor cells of CDC may be indistinguishable from them. But the nuclei of NET are round to oval and uniform in size, the mitotic figures are rare. The positive staining of pan-CK and neuroendocrine marker as CgA and Syn are helpful to make the right diagnosis.

The tumor cells of epithelioid angiomyolipoma (AML) are round to polygonal with abundant granular cytoplasm and enlarged vasicular nuclei often with prominent nucleoli, which may be initially misdiagnosed as a high grade carcinoma as CDC. Although almost all the epithelioid angiomyolipoma lack classical tri-phasic AML areas, it can be distinguished from CDC by immunohistochemical positive staining for Melan-A and HMB-45, and partly Actin [1].

Another non-epithelial tumors as renal pigmented paraganglioma and renal mesenchymal chondrosarcoma should be ruled out from the differential diagnosis. Renal pigmented paraganglioma [16] has the typical features of paraganglioma. The tumor cells were characteristically arranged in nests (“Zellballen”) or trabecular patterns bound by a delicate fibrovascular stroma. They are also large round or polygonal with round, oval, hyperchromatic nuclei. Approximately two-thirds of the tumor cells showed some degree of pigmentation. Prussian blue staining is useful to show the abundant iron in cytoplasmic. The tumor cells are positive for CD56 and CgA, which is helpful to distinguish from the CDC tumor cells.

Mesenchymal chondrosarcoma of kidney is a very rare tumor of kidney [17], which is consisted of undifferentiated spindle to oval shaped cells, with hyperchromatic nuclei and scanty cytoplasm mainly arranged in Ewing’s sarcoma-like, lamellar or hemangiopericytomalike patterns. This pattern might be confused with CDC. Mesenchymal chondrosarcoma has also other areas demonstrated well defined islands of cartilage, which is totally different from CDC. Herein, sufficient resection from the sample is essential. The lack of immunoreactivity for epithelial markers as cytokeratin AE1/E3, EMA and E-cadherin is the important marker for the differential diagnosis.

## Conclusions

Collecting duct carcinoma of Bellini of kidney is a rare but highly aggressive renal neoplasm arising from the distal portion of the nephron. The tumor cells of CDC may invade the vascular, leading to large area coagulative necrosis, which might be misdiagnosed as kidney infarct. On the other hand, the diagnosis of CDC should not be made before the exclusion the other kidney lesions.

## Consent

Written informed consent was obtained from the patient for publication of this Case Report and any accompanying images. A copy of the written consent is available for review by the Editor-in-Chief of this journal.

## Abbreviations

CDC: Collecting duct carcinoma; CCRCC: Clear cell renal cell carcinoma; NET: Neuroendocrine tumor; AML: Epithelioid angiomyolipoma; CT: Computed tomography.

## Competing interests

The authors declare that they have no competing interests.

## Authors’ contributions

QX carried out pathological examination, drafted the manuscript and performed the literature review; QC performed the literature review and collected the patient’s clinical information; NL carried out the immunohistochemical staining; ZF provided the radiological datas; ZY participated in pathological investigations and helped to draft the manuscript .TP gave and reviewed the final histopathological diagnosis, and revised the manuscript. All authors have read and approved the final manuscript.
